# Scope and Incentives for Risk Selection in Health Insurance Markets With Regulated Competition: A Conceptual Framework and International Comparison

**DOI:** 10.1177/10775587231222584

**Published:** 2024-01-29

**Authors:** Richard C. van Kleef, Mieke Reuser, Thomas G. McGuire, John Armstrong, Konstantin Beck, Shuli Brammli-Greenberg, Randall P. Ellis, Francesco Paolucci, Erik Schokkaert, Juergen Wasem

**Affiliations:** 1 Erasmus Centre for Health Economics Rotterdam (EsCHER), Erasmus University Rotterdam, The Netherlands; 2 National Institute for Public Health and the Environment, Bilthoven, The Netherlands; 3 Harvard Medical School, Boston, MA, USA; 4 University of Lucerne, Switzerland; 5 The Hebrew University of Jerusalem, Israel; 6 Boston University, MA, USA; 7 The University of Newcastle, Callaghan, NSW, Australia; 8 University of Leuven, Belgium; 9 University of Duisburg-Essen, Nordrhein-Westfalen, Germany

**Keywords:** health insurance, regulated competition, risk selection, international comparison

## Abstract

In health insurance markets with regulated competition, regulators face the challenge of preventing risk selection. This paper provides a framework for analyzing the scope (i.e., potential actions by insurers and consumers) and incentives for risk selection in such markets. Our approach consists of three steps. First, we describe four types of risk selection: (a) selection by consumers in and out of the market, (b) selection by consumers between high- and low-value plans, (c) selection by insurers via plan design, and (d) selection by insurers via other channels such as marketing, customer service, and supplementary insurance. In a second step, we develop a conceptual framework of how regulation and features of health insurance markets affect the scope and incentives for risk selection along these four dimensions. In a third step, we use this framework to compare nine health insurance markets with regulated competition in Australia, Europe, Israel, and the United States.

## Introduction

Over the past decades, more and more individual health insurance markets have been organized according to principles of “regulated competition.” The theory of regulated competition traces back to the early work by Alain Enthoven (1980) on “Consumer-Choice Health Plan” and has been further developed by Enthoven himself and other scholars (e.g., Enthoven, 1993; van de Ven et al., 2013). According to this theory, a well-designed combination of regulation and competition can enhance the fairness and efficiency of health care financing. Key aspects of this theory include a periodic choice of insurance plan by consumers (which generates competition among insurers), tools for insurers to improve the efficiency and quality of plans (e.g., selective contracting of providers, management of utilization, and choice of provider payment method), and a regulator (typically a government) that sets the rules of the game and manages competition.

Examples of health insurance markets with regulated competition include the national health insurance schemes in Belgium (until 2022), Germany, Israel, the Netherlands, and Switzerland; the voluntary health insurance markets in Australia and Ireland; and specific sectors, such as the state-based Marketplaces and Medicare Advantage, in the United States. Though each of these systems has a unique design, there are many commonalities, in terms of both how markets are regulated and the nature of competition. In all systems, regulators face the challenge of balancing regulation and competition in such a way that efficiency and fairness goals are achieved. When it comes to the design of regulated competition, however, there is no one-size-fits-all. Since specific objectives (and the weighting of these objectives) may differ across settings, the optimal design is likely to vary across settings too. As shown by McGuire and van Kleef (2018), both regulation and the nature of plan competition indeed vary substantially across countries. One important commonality, however, is the reliance on premium-rate restrictions to enhance the affordability of coverage for high-risk people and reduce reclassification risk. While these restrictions address important market failures, they exacerbate risk selection by consumers and insurers. Risk selection by consumers (sometimes referred to as adverse selection) includes selection in and out of the health insurance market and selection between high- and low-value plans. Risk selection by insurers (sometimes referred to as preferred-risk selection) includes actions to attract profitable consumers and deter unprofitable ones, for example, through the design and marketing of insurance plans. A long-standing literature explains how risk selection from either source threatens the functioning of health insurance markets (e.g., Akerlof, 1970; Cutler & Reber, 1998; Einav & Finkelstein, 2011; Feldman & Dowd, 1982; Glazer & McGuire, 2000; Newhouse, 1996; Rothschild & Stiglitz, 1976). For example, selection by low-risk people out of insurance pushes up average premiums, which undermines individual affordability of coverage and may create destabilizing upward premium (and downward enrollment) spirals. Selection through plan design (by insurers) can lead to inefficient restrictions on access and coverage for some conditions. Moreover, any investment by insurers in activities with an effect only on selection, such as advertising toward predictably profitable groups, has social costs but no social benefits.

Given that health insurance markets with regulated competition generally rely on premium-rate restrictions, *all* regulators face the challenge of mitigating risk selection. The nature and magnitude of selection problems, however, may differ across modalities of regulated competition. More specifically, the “scope” (i.e., the possible actions by consumers and insurers) and “incentives” for risk selection depend on relevant system features, such as the presence/absence of an insurance mandate, the degree to which coverage is standardized (in terms of benefits, cost sharing, and provider network), and the design of the payment system. For example, the “scope” for risk selection by insurers will be greater in systems with little standardization of coverage than in systems with high standardization of coverage. And “incentives” for selection by insurers might be greater in systems with risk equalization based on demographic information only than in systems that also risk adjust for health and socioeconomic variables. Selection by insurers will be more of a problem when both “scope” and “incentives” are present, and the same is true for selection by consumers. Nevertheless, the system features affecting the scope and incentives for selection by consumers might be different from those affecting the scope and incentives for selection by insurers. For example, risk equalization will reduce selection incentives for insurers, but not necessarily for consumers. The goal of this paper is to develop a conceptual framework of how health system features affect the scope and incentives for risk selection in health insurance markets with regulated competition. This framework might help regulators, policymakers, and researchers assess the potential for risk selection within modalities of regulated competition, given the features of these modalities.

The paper proceeds as follows. Section “New Contribution” highlights how this work provides new insights above and beyond existing literature. Section “Conceptual Framework” provides a conceptual framework describing different types of selection and how their magnitude is influenced by regulation and system features. As a next step, we apply our framework to the nine national and sub-national health insurance markets mentioned above. Section “Relevant Features of Nine Health Insurance Systems With Regulated Competition” summarizes the nine markets in terms of regulation and system features, and Section “Differences in Scope and Incentives for Selection Across the Nine Systems” compares the scope and incentives for each selection type across the nine systems. Finally, Section “Discussion” summarizes the key takeaways and policy implications.

## New Contribution

Although there is a vast literature on risk selection in regulated health insurance markets, there is no comprehensive framework of how system features affect the scope and incentives for risk selection in these markets. This paper develops such a framework. We start from the four types of risk selection distinguished by van Kleef, McGuire, et al. (2019): (a) selection by consumers in and out of the market, (b) selection by consumers between high- and low-value plans, (c) selection by insurers via plan design, and (d) selection by insurers via other channels. We go beyond earlier work by describing how system features affect the scope and incentives for each of these selection types. An important observation from our analysis is that system features affect the scope and incentives for the four selection types in different ways. As an additional step, we apply our framework to nine health insurance markets with regulated competition in Australia, Europe, Israel, and the United States. More specifically, we first map the relevant system features for each market and then compare the nine markets along the dimensions of scope and incentives. Our framework and system comparison help us understand why specific types of risk selection are more prevalent in some modalities of regulated competition than in others. In the “Discussion” section, we elaborate on the relevance and implications of our work for health policy and research.

## Conceptual Framework

In the literature, “risk selection” can refer to different types of actions. This section first defines what we mean by risk selection (Section “Risk Selection: A Definition”) and identifies four types of risk selection (Section “Four Types of Risk Selection”), building on van Kleef, McGuire, et al. (2019). After that, Section “How Do System Features Affect the Scope and Incentives for Risk Selection?” describes how features of the health insurance system affect the “scope” and “incentives” for each selection type.

### Risk Selection: A Definition

In the spirit of Newhouse (1996), we define risk selection as “actions by consumers and insurers to exploit unpriced risk heterogeneity” (emphasis added).^1,2^ “Actions” in this definition corresponds to our term, “scope,” and refers to what an insurer or consumer can do. “Unpriced risk heterogeneity” corresponds to our term “incentives.” Importantly, the concept of “unpriced risk heterogeneity” on the side of consumers can differ from that on the side of insurers (see Table 1). The relevance of this distinction is that system characteristics can influence unpriced risk heterogeneity in different ways. Community-rated premiums, for instance, increase unpriced risk heterogeneity on the side of consumers as well as on the side of insurers. This is distinct from risk equalization across insurers, which compensate for the unpriced risk heterogeneity on the side of insurers, but not for that on the side of consumers.

**Table 1.** Unpriced Risk Heterogeneity: Consumer Side Versus Insurer Side.

Side	Concept of “unpriced risk heterogeneity”
Consumer	*Unpriced risk heterogeneity* refers to the variation in gaps between consumers’ expectation of spending covered by an insurance contract and the net premium for that contract. By *net premium* for an insurance contract, we mean the gross insurance premium offered by the insurer minus any subsidy (e.g., in the form of a fixed subsidy, tax credit, or risk-adjusted voucher) received by consumers for obtaining that contract.
Insurer	*Unpriced risk heterogeneity* refers to the variation in gaps between insurers’ expectation of the costs of an insurance contract and the expected revenues associated with that contract. As we will discuss in this paper, the insurer’s revenues can consist of premiums and other features such as risk equalization and risk-sharing payments.

*Note.* Based on van Kleef et al. (2019).

As we will see in Section “Relevant Features of Nine Health Insurance Systems With Regulated Competition,” all nine systems in our country comparison, except the basic health insurance in Israel, rely on community-rating *per insurance plan* (and in some cases per insurance plan, age group, and/or region). In these systems, premiums can thus differ across insurance plans, implying that some of the unpriced risk heterogeneity can become priced, though indirectly, for example, when low-risk people and high-risk people sort into different plans.

### Four Types of Risk Selection

This section briefly describes the four types of selection as well as their potential effects on efficiency and fairness (Table 2).^3,4^ We assume that premiums are community-rated per insurance plan.

**Table 2.** Types of Risk Selection and Their (Potential) Effects.

Type of risk selection		(Potential) effects
Consumers	In and out of the market	• Inefficient uptake of basic coverage • Interferes with fairness objectives • (Risk of) market being adversely selected
	Between high- and low-value plans	• Inefficient sorting of consumers across plans • Interferes with fairness objectives • (Risk of) plans being adversely selected
Insurers	Via plan design	• Inefficient insurance plan design • Unavailability of high-quality care • Interferes with fairness objectives
	Via other channels	• Waste of resources • Interferes with fairness objectives • Suboptimal customer service • Negative spillover to supplementary plans

*Note.* Based on van Kleef et al. (2019).

#### Type 1: Selection by Consumers “In and Out of the Market”

A rational consumer bases his health insurance choice on the premium, the coverage (including network design, cost sharing, and other managed care features), his medical needs, and risk preferences. Given the substantial variation in expected medical needs, rational consumer behavior can lead to selection by some consumers “in and out of the market.” For example, low-risk people might not buy health insurance because the premiums for any plan exceed their expected medical spending covered by the insurance contract, even allowing for any “risk premium” the person might be willing to pay over and above the expected value of medical spending. In the presence of community rating and in the absence of government subsidies, premiums will be based on the mean expected insurer spending in the entire population. Such a flat premium typically exceeds the expected spending of low-risk people and falls below the expected spending of high-risk people. Consequently, insurance will be (much) more attractive for the second group than for the first, triggering selection in and out of the market. This type of selection can have several welfare effects. First, low-risk consumers who would have bought health insurance coverage for an actuarially fair premium may not buy when charged the higher community-rated premium (Geruso & Layton, 2017). Second, the intended cross-subsidies from low- to high-risk people might not be (fully) achieved, which might interfere with fairness objectives regarding equity in health care financing. Third, the dynamics of community-rated pricing may lead to upward premium spirals which further aggravate the aforementioned problems and undermine fairness objectives regarding individual affordability of coverage (Cutler & Reber, 1998). Fourth, if some individuals (either low risk or high risk) remain uninsured, they might not be able to afford (expensive) medical treatments in case of need, which might interfere with fairness objectives regarding equity in access to care. Fifth, the market as a whole faces a risk of being adversely selected, which might result in higher loading fees and market instability (e.g., due to bankruptcy of adversely selected plans).

#### Type 2: Selection by Consumers Between High- and Low-Value Insurance Plans

Selection by consumers can also take place *within* the market, for reasons similar to those just discussed for “selection in and out of the market.” When consumer preferences are correlated with expected medical spending under an insurance contract, different risk types tend to sort into different plans (Bundorf et al., 2012). For instance, high-risk people might be more likely to sort into more generous coverage (e.g., a low-deductible plan) than low-risk people. This type of selection can have several welfare effects. First, it distorts the incremental premium of the more generous plan. More specifically, as an outcome of selection by consumers across plans, the incremental premium of the generous plan does not only reflect variation in plan value but also the effect of selection. This may lead too few people to choose that plan (Einav & Finkelstein, 2011).^5^ Second, and related to the first effect, the inefficiently elevated price for the more generous plan embodies a redistribution benefiting the healthier types (whose premium is lowered by the selection) at the expense of the sicker types (whose premiums are elevated). Societies might consider such redistribution unfair. Third, insurance plans face a risk of being adversely selected, which might result in higher loading fees and market instability.

#### Type 3: Selection by Insurers via Plan Design

To the extent that insurers care about profits and losses, variation in expected profitability of individual insurance contracts (i.e., variation in gaps between expected costs and revenues) comes with incentives for risk selection. Though regulated health insurance markets typically require open enrollment, insurers can engage in other types of risk selection, sometimes referred to as indirect selection (Breyer et al., 2011; van de Ven & Ellis, 2000). High-risk people are likely to be different than low-risk people in terms of their patterns of health care use; for example, high-risk people may suffer relatively more from certain chronic illnesses. In this case, an insurer can design its insurance plan(s) in a way to appeal differentially to these groups (Ellis & McGuire, 2007; Glazer & McGuire, 2000; Layton et al., 2017). Examples include not contracting providers that are particularly attractive to people with specific conditions (Shepard, 2022) and setting high (low) copayments for drugs specifically used by unprofitable (profitable) people (Carey, 2017; Geruso et al., 2019; Han & Lavetti, 2017; Lavetti & Simon, 2018). Other type-3 actions include the use of waiting times and authorizations. Risk selection by insurers threatens efficiency by distorting the form of plan offerings. Ideally, a competitive insurance market incentivizes insurers to design plans to serve consumer preferences, rather than designing plans in response to selection incentives. Selection via plan design not only reduces the efficiency of plan offerings but can also distort the availability of high-quality care, particularly for high-risk people, the ones insurers most want to avoid (van de Ven et al., 2015). The reason is that when insurance plans do not cover high-quality care for services predominantly used by unprofitable consumers, health care providers face disincentives to offer and invest in these services. Note, however, that the tools that insurers can use to affect selection (e.g., drug formulary placement and selective contracting) are the very tools insurers use to control costs and/or to improve the quality of the care covered, making effective regulation of these tactics particularly difficult. To the extent that selection actions by insurers are successful (i.e., lead to sorting of risk types into different plans), these actions can result in premiums that are lower for low-risk people than for high-risk people, which can conflict with fairness (see also type-2 selection by consumers).

#### Type 4: Selection by Insurers via Other Channels

In addition to selection via plan design, insurers can also engage in other types of selection actions, for example, regarding marketing, customer service, provider incentives, and tie-in sales. For example, insurers might use longer query-response times for people from high-cost regions than from low-cost regions (Bauhoff, 2012). In addition, insurers might instruct/incentivize health care providers to encourage expensive patients to switch to another insurer or plan type.^6^ Marketing actions might include selective advertisement and/or tie-in sales to appeal to active, more healthy enrollees. For example, insurers might direct advertising to young and healthy communities. Tie-in sales could mean that insurers price and/or design supplementary insurance plans in a way that these are relatively (un)attractive to people who are (un)profitable for the basic plan. Moreover, insurers could simply not offer supplementary insurance plans to unprofitable groups. To the extent that risk selection leads to the sorting of risk types into different plans, these actions can conflict with fairness objectives (see also type-2 selection by consumers). Moreover, from a societal perspective, resources used only for risk selection are wasted since this type of risk selection by insurers is a zero-sum game. In the case of selection via pricing, design, and/or underwriting of supplementary insurance plans, risk selection can threaten the affordability, accessibility, and efficiency of supplementary plans.

### How Do System Features Affect the Scope and Incentives for Risk Selection?

The importance of any of the four potential selection problems will depend on the specific features of a health insurance market. For example, the in-and-out-of-the-market problem is essentially non-existent in the *mandatory* insurance systems in Belgium, Israel, the Netherlands, and Switzerland, but one of the most significant issues in the *voluntary* systems in Australia, Ireland, and the U.S. Marketplaces.^7^ These and other differences in the institutional contexts that affect selection scope and/or incentives emerge in our analyses below.

In terms of scope, we identify nine important characteristics, summarized in Table 3. An insurance mandate obligates consumers to obtain health insurance coverage. Obviously, the strength of such a mandate depends on the consequences of disregarding it, such as encountering a tax penalty for not buying insurance.^8^ Standardization of contract length and switching options refer to the length of the insurance contract period. As we will see later, regulators typically specify 1-year contracts, meaning that consumers can switch insurer (or insurance plan) every 12 months. By standardization of benefits, consumer cost sharing, provider network, and coverage for out-of-network spending, we mean the extent to which these aspects are determined by law, limiting or eliminating discretion by insurers on these dimensions of coverage. Countries differ, but regulators typically rely on extensive standardization, limiting insurer flexibility. By regulation of marketing and customer service, we mean, for example, restrictions applied by the regulator with respect to welcome presents and required response to enrollment inquiries. Forms of advertising may be difficult to regulate. The link between insurers and providers of care refers to how effectively an insurer can transmit its incentives (to, e.g., discourage enrollment of some type of patients) to providers. Such transmission might be through the choice of providers for inclusion in a network or by “performance” metrics built into payment contracts. Coverage and regulation of supplementary insurance refers to the benefits covered by supplementary insurance and obligations in terms of standardization and pricing. Note that a prohibition on tie-in sales might not be effective in avoiding selection via supplementary plans. In the case of the Netherlands, for instance, basic and supplementary insurance plans are contractually separated by law. Nevertheless, consumers tend to perceive the two products as one (Duijmelinck & van de Ven, 2014). This implies that any flexibility in terms of design, pricing, and underwriting regarding supplementary insurance provides insurers with a powerful tool to select in favor (against) consumers who are expected to be (un)profitable for the basic health insurance. Table 3 summarizes how these nine features can influence the scope for each type of selection.

**Table 3.** Main Effects of System Features on the Scope for Risk Selection.

System feature	Selection by consumers		Selection by insurers	
	In and out of basic coverage	Between high- and low-value plans	Via plan design	Via other channels
**Insurance mandate**	A (strong) mandate eliminates the possibility for consumers to select out of coverage in response to health events	—	—	—
**Standardization of contract length and switching options**	Longer contracts reduce the possibilities for consumers to change insurance coverage/plans in response to health events		Standardization of contract length reduces the possibilities for insurers to induce advantageous selection	—
**Standardization of benefits**	—	Standardization of benefits, cost sharing, provider network, and out-of-network spending reduces the “contract space” of insurance plans. More specifically, standardization reduces the variation in plan quality, which mitigates the scope for selection between high- and low-value plans. Moreover, it mitigates the possibilities for insurers to attract (deter) profitable (unprofitable) people via plan design.		—
**Standardization of consumer cost sharing**	—			—
**Standardization of provider networks**	—			—
**Standardization of coverage for out-of-network spending**	—			—
**Regulation of marketing and customer service**	—	—		Restrictions on marketing and customer service reduce possibilities for insurers to engage in risk selection.
**Link between insurers and providers of care**	—	—		A stronger link between insurers and providers of care increases scope for selection via healthcare providers
**Regulation of supplementary insurance**	—	—		Restrictions on the design, pricing, and underwriting of supplementary plans reduce possibilities for insurers to use supplementary plans as a selection tool for the basic plan.

In terms of the incentives for risk selection, we also identify nine important system features (see Table 4). By premium-rate restrictions, we mean limitations on insurers’ differentiation of their premiums to (specific) individual characteristics. Regulators typically strictly limit risk rating, in the extreme requiring community rating per insurance plan. As discussed above, rate restrictions are a fundamental cause of selection incentives. To evaluate the effects of the other eight system features in Table 4, we assume the presence of premium-rate restrictions as well as provisions ensuring open enrollment (an insurer must accept every applicant).

**Table 4.** Main Effects of System Characteristics on the Incentives for Risk Selection.

System feature	Selection by consumers		Selection by insurers	
	In and out of coverage	Between high- and low-value plans	Via plan design	Via other channels
**Premium-rate restrictions**	Premium-rate restrictions increase unpriced risk heterogeneity, both on the side of consumers and that of insurers.			
**Observable diversity of the population covered** ^a^	A more diverse population comes with more variation in expected spending and thereby increases unpriced risk heterogeneity, both on the side of consumers and that of insurers.			
**Breadth of coverage** ^a^	In general, more comprehensive coverage comes with larger differences in expected spending under insurance contracts between low- and high-risk people and thereby increases unpriced risk heterogeneity, both on the side of consumers and that of insurers.			
**Depth of coverage** ^a^				
**Risk equalization** ^a^	To the extent that risk equalization/sharing payments are financed internally via plan premiums, they might elevate premiums of plans preferred by low-risk people (e.g., high-deductible plans) and thereby increase incentives for selection out of the market.	Risk equalization and risk sharing mitigate selection-driven premium variation between high- and low-value plans and thereby mitigate incentives for selection by consumers between high- and low-value plans.	Risk equalization and risk sharing compensate insurers for variation in (expected) costs of insurance contracts and thereby mitigate incentives for selection by insurers.	
**Risk sharing** ^a^				
**External financing of risk equalization/sharing fund** ^a,b^	To the extent that subsidies to consumers or insurers are financed with external resources (e.g., tax revenues), (net) insurance premiums will be lower which—for a given level of willingness to pay—mitigates selection out of the market.	—	—	
**Subsidies to consumers** ^a^		To the extent that subsidies are based on premiums they reduce variation in net premiums and thus mitigate selection between high- and low-value plans.	—	—
**Organizational form of insurance companies** ^a^	—	—	For-profit insurers might be more sensitive to predictable profits and losses than non-profit insurers.	

a For these effects, we assume community-rated premiums and open enrollment.

b Note that this feature is only about “how the risk equalization/sharing fund is filled” (and not about “how the money in the fund is allocated to insurers”).

Observable diversity of the population covered refers to the variation of observable individual characteristics (such as age, health, and socioeconomic status) connected to health care costs within the total pool of enrollees. In mandatory schemes, populations might be more diverse than in voluntary schemes. Scope and depth of coverage refer to the benefits covered and the level of cost sharing, respectively. Risk equalization refers to the regulator’s formula for compensation of insurers for variation in *expected* spending across contracts. For example, insurers might receive higher payments for covering the elderly and chronically ill than for covering the young and healthy. Risk sharing means that insurers receive some revenue related to *actual* spending during the contract year. For example, insurers might receive a cost-based compensation for individual-level spending above a certain threshold (sometimes referred to as reinsurance, outlier-risk sharing, or excess-loss compensation). By external financing of the risk equalization/sharing fund, we mean the extent to which outside sources, typically the government, contribute resources to the funds to be distributed. As will be shown later, some countries rely more on internal contributions (i.e., via insurance premiums), while others rely more on external financing (e.g., general tax revenues). Consumer subsidies refer to vouchers, tax credits, and other subsidies. Finally, incentives for risk selection (or the response to these incentives) might be affected by the organizational form of insurance companies.^9^ Table 4 summarizes how these nine system characteristics can influence the incentives for each type of selection.

## Relevant Features of Nine Health Insurance Systems With Regulated Competition

Our international comparison includes nine health insurance systems with regulated competition: the mandatory health insurance schemes in Belgium, Germany, Israel, the Netherlands, and Switzerland, the state-based Marketplaces and Medicare Advantage market in the United States, and the voluntary health insurance markets in Ireland and Australia. Although a full description of these systems is beyond the scope of this paper, this chapter summarizes their most important features. More detailed descriptions can be found in McGuire and van Kleef (2018). An important commonality of the nine systems is that they rely on a combination of regulation and competition to enhance fairness and efficiency. In all nine systems, consumers have a free choice of insurance plan and insurers compete on price (except Israel) and quality. A government sets the rules of the game and manages competition. The degree of competition varies across systems.

The mandatory health insurance in *Belgium* provides coverage for a comprehensive package of care. Spending under this scheme is almost entirely financed with external resources coming from social security contributions and taxes; the community-rated premium that consumers pay to their insurer directly is very low. In recent years, the premium has fallen to zero. Although the system includes elements of regulated competition, insurers have few tools to improve the efficiency of care since coverage is completely standardized, both in terms of benefits covered and level of cost sharing. Insurers negotiate as a cartel with providers on budgets and prices. Although insurers have no flexibility regarding plan design, risk selection is possible via marketing and supplementary plans. All insurers participate in a payment system that combines sophisticated risk equalization with proportional risk sharing (Schokkaert et al., 2018).^10^

The so-called “sickness fund insurance” in *Germany* is mandatory for employees below a certain income threshold and retired employees, a group that represents about 90% of the population. The remaining 10% (employees above the income threshold, self-employed, and civil servants) can choose between sickness fund insurance and private health insurance (PHI) (with risk-rated premiums). Sickness funds are financially responsible and compete for enrollees who can switch funds once a year. Enrollees pay an income-related premium composed of two parts: a uniform contribution of about 15% and a fund-specific contribution that varies between 0.3% and 1.8% (2017). Sickness fund plans are highly standardized. Nevertheless, sickness funds can offer voluntary deductibles and have some flexibility in terms of contracting. In practice, however, most of the provider contracting is done by sickness funds collectively. Sickness funds are obliged to participate in a sophisticated risk equalization system that is supplemented with 80% risk sharing of individual-level spending above 100,000 euros per year (Wasem et al., 2018).

*Israel*’s basic health insurance is mandatory for the entire population and provides comprehensive coverage. Spending under this scheme is externally financed with income-related taxes; consumers do not pay a premium to their insurer. This implies that insurers fully depend on payments from the government which are allocated through a simple risk equalization model. While benefits and consumer cost-sharing schedules are highly standardized, insurers have substantial flexibility regarding provider network design. Three insurers even own hospitals, a form of vertical integration. This all implies that, from the consumers’ perspective, plans mainly differ in terms of provider network and managed care (Brammli-Greenberg et al., 2018).

The basic health insurance in the *Netherlands* is mandatory for the entire population and is executed by private insurers with financial responsibility. About 50% of total spending is financed by community-rated premiums for people ≥18 complemented by a government subsidy for people <18. The other 50% is financed by income-related tax contributions. The main dimensions in which plans differ include deductible level, provider network, and copayments for out-of-network spending. All insurers participate in a sophisticated risk equalization scheme that is supplemented with some outlier-risk sharing for mental care. Insurers negotiate with providers on budgets, prices, and volumes. Vertical integration is allowed, but hardly found in practice (so far). All insurers offer supplementary plans on top of the basic plan. Although the two plan types are contractually separated by law, almost all people with a supplementary plan buy their basic plan from the same insurer (van Kleef et al., 2018).

*Switzerland*’s mandatory health insurance provides coverage for a broad package of care. Spending is financed by out-of-pocket premiums (which are community-rated per plan, region, and age group) and cantonal payments for about half of inpatient hospital costs, a form of risk sharing. Apart from the cantonal payments and copayments for consumers, insurers are financially responsible for spending under the benefits package. A moderately sophisticated risk equalization system is in place to redistribute premium revenues from insurers with disproportionate shares of young and healthy people to those with disproportionate shares of elderly and chronically ill people. This redistribution, however, occurs within each of the 26 cantons but not across these cantons. Consumers have a choice of deductible level and plan type (e.g., standard plan with free access to providers vs. managed care plans with gatekeeping) and can switch plans twice a year. Insurers have substantial flexibility when it comes to the marketing of insurance plans and the design of managed care plans (Schmid et al., 2018).

The *U.S. Marketplaces* are intended to provide coverage to those who do not receive insurance through their employer or through public programs. Although the Patient Protection and Affordable Care Act imposed penalties for absence of insurance, financial penalties have been removed, making enrollment in a Marketplace plan a voluntary decision. (Some U.S. states mandate insurance coverage and penalize noncompliance.) Spending under this scheme is financed internally by enrollee premiums that can vary by geography and age but not health status. Low-income people are eligible for premium and cost-sharing subsidies. Insurers participate in a sophisticated risk equalization scheme with some outlier-risk sharing for very high spenders effected through the transfer formula among insurers in a state. Insurance plans are designated by “metal levels” bronze, silver, gold, and platinum and can substantially differ in terms of cost sharing and provider network. Consequently, consumers have a broad set of choice options and insurers have substantial flexibility regarding plan design (Layton et al., 2018).

*U.S. Medicare Advantage* is the private alternative to Traditional Medicare, a public scheme for people of 65 years and older and those with serious chronic diseases. Traditional Medicare coverage is composed of Part A for hospital care, Part B for medical services, and Part D for drugs. Enrollment in Part A is automatic for eligible individuals and requires no premium. Premiums for Part B are set by regulation and go up by the income of the beneficiary. Premiums for Part D are community-rated within plans. Spending under Part A is financed by the federal government, and premiums for Parts B and D are highly subsidized. Medicare Advantage plans differ across a wide spectrum of cost-sharing schedules, provider network, and coverage of out-of-network spending (but are obliged to offer the same or better actuarial value than Part A and B coverage in Traditional Medicare). Consequently, consumers have many choice options and insurers have substantial flexibility regarding plan design. Medicare Advantage is offered by private, commercial insurers who must respect Medicare’s regulation, for example, regarding bidding and participation in a sophisticated risk equalization system (McGuire & Newhouse, 2018).

*Australia*’s PHI exists next to two public systems: a nation-wide program called Medicare and seven State and Territory-level health care systems. PHI provides a combination of duplicative coverage (of public hospital care delivered by States and Territories), substitute coverage (for care not covered by the public systems, for example, private care delivered in private hospitals), and supplementary coverage (for services not covered by the public systems). From a consumer’s perspective, the main benefits of having PHI include a free choice of doctor, shorter waiting times, some supplementary coverage, and avoidance of tax penalties for not having PHI. With the aim to mitigate the pressure on public finances, the Australian government applies specific regulations to PHI, including requirements of open enrollment and community-rating per insurer per plan. In addition, the government has implemented some “sticks and carrots” in the form of penalties (rewards) for delayed (early) enrollment to promote coverage among young people, tax discounts to promote PHI uptake among high-income people, and subsidies to support low-income individuals. PHI coverage is standardized to a limited degree, resulting in substantial product differentiation. Consequently, consumers have many choice options and insurers have significant flexibility in plan design. All insurers participate in a claims-equalization system that redistributes medical spending via a combination of proportional risk sharing (with risk shares varying by age) and high-cost outlier-risk sharing. Insurers are financially responsible for all spending net of risk sharing. At present, there is no prospective risk adjustment of plan payment in Australia (Paolucci et al., 2018).

Like Australia, *Ireland* has a voluntary PHI that exists in parallel with a public system. The Irish government has implemented specific regulation to enhance the accessibility and affordability of PHI plans including a requirement of open enrollment and premium-rate restrictions. Although PHI mainly covers hospital care, insurers are free to include additional benefits. Most of the coverage is duplicative of the public scheme. Motives for consumers to take up PHI include faster access and access to more luxury hospitals. Spending under PHI is financed by out-of-pocket premiums that are community-rated per plan and per age group. To promote uptake of voluntary insurance the government has implemented a system of tax reliefs. Plans differ in terms of additional benefits covered, cost sharing, and provider network. Consequently, consumers have many choice options and insurers have substantial flexibility regarding plan design. Insurers are obliged to participate in a simple risk equalization scheme with some (implicit) elements of risk sharing (Armstrong, 2018).

Tables 5 and 6 summarize the nine systems in more detail, according to the features presented in Tables 3 and 4, respectively. Together with the system descriptions above, Tables 5 and 6 will form the basis for our comparison of scope and incentives for risk selection in the next section.

**Table 5.** System Characteristics Affecting the Scope for Risk Selection.

System feature	Belgium	Germany	Israel	Netherlands	Switzerland	U.S. Marketplaces	U.S. Medicare Advantage	Australia	Ireland
**Insurance mandate**	YES	YES (Certain groups can choose between social and private health insurance.)	YES	YES	YES	NO (Penalties for nonpurchase were removed.) ^a^	NO (Although everyone over age 65 is eligible for Part A basic coverage for no premium.)	NO (However, tax surcharges for high-income people who are not enrolled and 2% premium loadings for those who purchase PHI after their 31st birthday.)	NO
**Standardization of contract length and switching options**	HIGH Consumers can switch every 3 months.	HIGH Consumers can switch every 12 months.	HIGH Members can switch twice a year.	HIGH Consumers can switch every 12 months.	HIGH Members can switch twice a year (with some exemptions)	HIGH Consumers can switch every 12 months.	HIGH Consumers can switch every 12 months.	LOW No minimum duration and switching at any time.	HIGH Consumers can switch every 12 months.
**Standardization of benefits**	HIGH Fixed benefits package.	HIGH More than 97% of expenditures are within a fixed package.	HIGH Fixed benefits package.	HIGH Fixed benefits package.	HIGH (Fixed package that differs only for some new and expensive rare disease pharmaceuticals.)	HIGH Fixed benefits package.	MODERATE Medicare advantage has tiers of covered services, as do optional supplementary plans. Insurers can cover additional benefits.	LOW/MODERATE Minimum benefit package of specific hospital treatments (basic plans). No maximum requirements regarding coverage of higher metal levels.	MODERATE A set of minimum benefits apply but insurers can provide (and actually compete on) additional benefits.
**Standardization of consumer cost sharing**	HIGH Complete standardization	MODERATE Insurers are allowed to offer voluntary deductibles to a limited extent. However, in practice, most do not.	HIGH Cost-sharing schedules are highly regulated in terms of the maximum amount that could be charged per service, the maximum of total copayments, exemptions, and discounts.	MODERATE Voluntary deductibles up to 500 euros p.p. per year; insurers can waive out-of-pocket spending under the mandatory deductible when consumers go to “preferred” providers.	MODERATE Voluntary deductibles of up to 2,200 CHF (on top of the mandatory deductible of 300 CHF) per person per year, while 10% copayment of up to CHF 700 is standardized.	LOW Can be a wide range of cost sharing from very little/none to 40% of total costs.	LOW Insurers are free to set cost-sharing schedules, subject to the constraint that the plan’s actuarial value equals or exceeds that of Traditional Medicare.	LOW Cost sharing varies significantly across plans.	LOW Health plans can choose their level of deductible to apply on any product and consumers have a choice of deductibles.
**Standardization of provider networks**	HIGH Selective contracting is not allowed.	MODERATE/HIGH Although there are 11,000 selective contracts, more than 95% of expenditures are within standardized collective contracts (between insurer associations and provider associations).	LOW Plans have high flexibility in terms of provider network design under the basic insurance.	LOW High flexibility, some requirements regarding travel/waiting time.	LOW In addition to the standard plan (which requires free choice of healthcare providers), insurers have the option to offer managed care plans with gatekeeping).	LOW Some regulation of doubtful effectiveness.	LOW Plans have high flexibility in terms of provider networks.	LOW Insurers have the freedom to contract selectively. However, services and providers covered must meet the quality assurance requirements.	NONE Health plans compete on the basis of provider access through selective contracting. In practice, most plans provide access to nearly all providers.
**Standardization of coverage for out-of-network spending (regarding services included in the benefits package)**	NOT APPLICABLE	HIGH No financial sanctions for out-of-network-spending for the insured.	NONE There is no coverage of out-of-network spending. Member who wants to go out-of-network can do it only privately or using voluntary health insurance.	MODERATE Out-of-pocket payment for out-of-network care should not be a “financial hurdle” for patients.	LOW Out-of-pocket payment for out-of-network spending is the standard; however, insurers apply this rule differently.	LOW While insurers are free to set coverage levels for out-of-network spending, there is regulation to prevent “surprise billing.”	LOW Insurers are free to set coverage levels for out-of-network spending.	LOW Agreement (e.g., no gap agreement, or known gap) and no agreement can take place for hospital coverage. Insurers may establish preferred provider arrangement for general treatment which may lead to reduced out-of-pocket spending.	NONE If a provider is not covered by a plan, no or very limited obligations apply.
**Regulation of marketing and customer service**	MODERATE Marketing of insurers is only moderately regulated.	MODERATE There are guidelines by the Federal Social Office and respective regional offices.	MODERATE Regulation of each plan of total spending on marketing. There are unsuccessful attempts to regulate plans that pull doctors and enrollees from other plans.	MODERATE Tie-in sales are forbidden, but insurers have a lot of flexibility regarding marketing and customer service.	MODERATE Marketing of insurers is only moderately regulated, however increased regulation for brokers.	MODERATE Websites facilitate choice and provide standardized information. State-level marketplaces vary.	MODERATE Government regulation of marketing of MA plans, but not customer service. Health plan report cards and star ratings are available.	WEAK Marketing of insurance plans is hardly regulated. Some privately owned websites serve as comparison tools.	MODERATE All insurance products must adhere to the Consumer Protection Code.
**Link between insurers and providers of care**	WEAK No strong links between insurers and providers. Some sickness funds have ties with pharmacies and/or outpatient clinics.	MODERATE Vertical integration is not allowed. Insurers have moderate contracting freedom regarding prices/payments and volumes	MODERATE/STRONG Three out of four insurers own hospitals; all insurers have providers as salaried employees.	MODERATE Vertical integration is allowed but hardly found in practice; insurers have substantial contracting freedom regarding price, quality, and volume of care.	WEAK Insurers have been owners of Managed Care providers, but today, this as any other link is rarely the case.	STRONG Some provider groups organize to provide insurance.	MODERATE High in some Medicare advantage plans with selective contracting. Weak overall.	STRONG No prohibition or allowance of vertical integration. Some insurers own dental care practices (for example).	MODERATE Mostly insurers negotiate with providers who are separate entities. Some vertical integration regarding primary care and diagnostic services.
**Link between basic and supplementary health insurance plans**	STRONG When consumers buy a supplementary plan from a sickness fund, they must also buy the basic plan from that fund.	WEAK Only private insurers can sell supplemental health insurance plans; sickness funds can co-operate with them regarding design of benefit schemes and marketing	MODERATE/STRONG Consumers must buy supplementary plans from the same insurer as their basic plan The regulation enforces separate administrative management.	STRONG Regulation that forbids tie-in sales is ineffective since 99% of people with supplementary insurance buy the basic plan from the same insurer.	STRONG Most insurers offer basic and supplementary insurance. However, the regulator requires a strict division into two financially separated plans.	NOT APPLICABLE Rare to have supplemental coverage; no link between the two.	NOT APPLICABLE Very rare to have supplementary coverage; no link between the two.	STRONG Tie-in sales are per se not regulated, and it is very unlikely to find consumers with products from different insurers.	NOT APPLICABLE All products must meet minimum benefit requirements so cannot be seen as supplementary.

a For firms with more than 50 employees, there is still an employer mandate to offer insurance plans. There are no penalties, however, for employees who do not enroll.

**Table 6.** System Characteristics Affecting the Incentives for Selection.

System feature	Belgium	Germany	Israel	Netherlands	Switzerland	U.S. Marketplaces	U.S. Medicare Advantage	Australia	Ireland
**Premium-rate restrictions**	Community rating per insurer, with very low individual premium.	Community rating per insurer (i.e., a community-rated percentage of income consisting of two parts: a uniform contribution of about 15% and a fund-specific contribution varying from 0.3% to 1.8%).	No out-of-pocket premium (public insurance is fully financed through a dedicated health insurance tax by the National Insurance Institute and general taxes; the health insurance tax amount is income-related and does not relate to plan membership).	Community rating per insurance plan and per deductible level.	Community rating per insurance plan, per voluntary deductible, per age group (0–18, 19–25, and 26+), per region, and per plan type (e.g., standard vs. managed care).	Community rating per insurance plan, per geographical region, and age category (with the restriction that the premiums cannot differ more than three-fold among categories).	Community-rated per insurance plan (consumer premiums over risk-adjusted federal contribution determined by plan bidding).	Community rating per insurance plan, age-based premium discounts for those 18–29.	Community rating per insurance plan, per age group (e.g., adults, children); possibility of some discounts up to a 10% of the central rate; lifetime community-rating requirements.
**Diversity of the population covered**	HIGHLY DIVERSE The entire population is covered.	HIGHLY DIVERSE About 90% of population covered.	HIGHLY DIVERSE The entire population is covered.	HIGHLY DIVERSE The entire population is covered.	HIGHLY DIVERSE The entire population is covered.	MODERATELY DIVERSE Old people not included, very sick/poor might move to Medicaid.	MODERATELY DIVERSE Only over age 65 and those with serious chronic conditions.	MODERATELY DIVERSE PHI is open to all. 48% of the Australian population is currently enrolled. Enrolled population is relatively wealthy.	MODERATELY DIVERSE PHI is open to all. 48% of the Irish population currently enrolled. Enrolled population is relatively wealthy.
**Breadth of coverage**	VERY LARGE Including hospital care, pharmaceutical care, primary care, and ambulatory care, but also some dental and nursing care and medical devices.	VERY LARGE Including hospital care, pharmaceutical care, medical devices, primary care, and ambulatory care, but also some dental and nursing care.	VERY LARGE Including hospital care, pharmaceutical care, medical devices, primary care ambulatory care, mental health care, emergency care, and some dental care.	VERY LARGE Including hospital care, pharmaceutical care, medical devices, primary care, and ambulatory care, but also some dental and nursing care.	VERY LARGE Including hospital care, pharmaceutical care, medical devices, primary and ambulatory care, and medical treatment of long-term care patients.	VERY LARGE Including hospital care, pharmaceutical care, medical devices, primary care, and ambulatory care.	LARGE Broad medical coverage, but pharmaceutical coverage is optional to plan. No dental, vision, or hearing coverage.	MODERATE Some healthcare services covered by Medicare are not covered by PHI.	MODERATE Coverage predominantly is for hospital care.
**Depth of coverage**	DEEP But rather large copayments. Moreover, balance billing in single hospital rooms and in ambulatory care.	VERY DEEP Annual co-pays are not more than 1%–2% of annual income.	VERY DEEP Copayment for specialist visits (~5 euro per visit per quarter; 15% copayment for prescribed drugs; no copayment for inpatient hospital care).	VERY DEEP Mandatory deductible of only 385 euro p.p. per year; some copayments for specific services.	VERY DEEP Mandatory deductible of only 300 euro p.p. per year and 10% copayment up to 700 euro per adult per year.	DEEP Front-end cost sharing can be high; high costs well-covered.	MODERATE Substantial out-of-pocket costs in most MA plans. Consumer choice and employer subsidies to retirees of more generous coverage allowed.	MODERATE Average gap payment when the gap was paid of $185.3 AUD (Dec 2020) for in-hospital care.	VERY DEEP Cost sharing represents no more than 5% of total spending.
**Risk equalization**	SOPHISTICATED Risk adjustors based on age, gender, socioeconomic information, prior-year use of pharmaceuticals, and prior-year DRGs; payment weights are not regularly updated.	SOPHISTICATED Risk adjustors based on age/gender, prior-year diagnosis, prior-year use of pharmaceuticals, and regional characteristics.	VERY SIMPLE Risk adjustors include age, gender, and place of residence (periphery vs. center). Separate prospective per-capita payments for five severe disease patients.	SOPHISTICATED Risk adjustors based on age/gender, socioeconomic information, multiple-year historical costs, and prior-year use of hospital care, pharmaceutical care, medical devices, and physiotherapy.	MODERATE Risk adjustors based on age/gender, canton (= region), prior-year use of hospital care and pharmaceutical care; 50% solidarity reduction for young adults (19–25).	SOPHISTICATED Risk adjustors based on age, current-year utilization, and diagnoses. Works in conjunction with complicated “transfer formula” that includes premium, geography, and high-cost spending factors.	SOPHISTICATED CMS-HCC model uses prior-year diagnoses and 11 clusters of pharmaceuticals together with flags for Medicaid eligibility and age-sex. Formula multiplies risk scores by a county-specific average payment.	NO The only prospective component is through premium payments.	SIMPLE Risk adjustors include age, gender, and type of cover (to distinguish between basic and more sophisticated products).
**Risk sharing**	LARGE Complicated formula. Everything taken together, insurers are responsible for only 7.5% of expenditures for healthcare services covered under the insurance scheme.	LIMITED 80% of annual individual-level spending above a threshold of 100k Euro is compensated by a high-cost pool.	LIMITED Between 10% and 15% from plan total budget distribute between the plans that achieved goals set by the state.	LIMITED Cost-based compensation of 90% for individual-level mental healthcare costs above about 90.000 euros.	LARGE 55% of individual inpatient costs are paid by cantons.	VERY LIMITED Confined to 60% above annual spending above $1m. Embedded in transfer formula	NONE No risk sharing. However, the formula increases payments as risk scores increase, hence protection against increases in apparent severity.	MODERATE Insurers participate in a scheme that combines proportional risk sharing (with risk shares varying by age) and outlier-risk sharing (82% of spending above $50,000 net of proportional risk sharing).	MODERATE A high-cost claims pool (HCCP) operates under which a compensation of 40% is given for the cost of claims in excess of €50,000 and other credits.^a^
**External financing of risk equalization / sharing fund**	YES The bulk of spending under the insurance scheme is financed externally by social security contribution and taxes; the annual premium is less than 100 euros per person per year and has been 0 in recent years.	YES 5.5 % of total expenditures (2019) are not financed by contributions of insured (or their employers) but from general tax money.	Yes About 40% of total spending is financed by general tax revenues. The remaining 60% is collected through a dedicated health insurance tax by the National Insurance Institute.	YES About 50% of total spending is financed via an out-of-pocket premium by people >17 and a government subsidy for people <18; the other part is financed with external funds coming from income-related contributions.	YES 55% of individual inpatient costs are paid by cantons.	NO Marketplaces self-funded. Individuals eligible for premium subsidies.	YES No fixed pool divided up. Hence severity increase fully accommodated by the Federal budget.	NO	NO The risk equalization/risk-sharing mechanism is self-funded though facilitated through the taxation system.
**Subsidies to consumers**	NO Due to external financing, premiums are already very low.	NO (Premiums are income-related)	NO (Premiums are income-related)	YES For about 50% of the population up to 90% of the average premium (in addition to income-related contributions).	YES About a quarter (27.5% in 2020) of the enrolled population receives a premium subsidy (of on average 47.9% in 2020).	YES High for low-income groups. Premium subsidies, cost-sharing subsidies.	YES Low-income enrollees can optionally have premiums and out-of-pocket coverage covered by Medicaid program.	YES Up to 32.8% of the premium for those 70+ with low income (2019–2020).	YES Tax reliefs are provided to encourage the take-up of health insurance.
**Organizational form of insurance companies**	Not-for-profit, entry forbidden.	Not-for-profit self-governing bodies of public law. The CEOs have some incentives to expand which translates into incentives for risk selection.	Not-for-profit, entry forbidden.	Not-for-profit by law, but all are private, risk-bearing companies. Moreover, many insurers are for-profit regarding supplementary insurance.	Not-for-profit by law (but many insurers are as well for-profit insurers in their supplementary insurance).	A mix of for-profit and not-for-profit; usually not specializing in Marketplaces; cooperatives permitted but most failed and left.	A mix of for-profit and not-for-profit. Almost always with a commercial enrollment. Originally plans were required to have a majority of privately insured enrollees.	A mix of for-profit and not-for-profit insurers.	A mix of for-profit and not-for-profit. The largest insurer is a state organization governed under parliamentary statute.

a In addition, a utilization credit is provided based on the length of the treatment received, and an over-compensation validation mechanism applies to ensure that recipient plans are not being rewarded excessively.

## Differences in Scope and Incentives for Selection Across the Nine Systems

**Figure 1.**
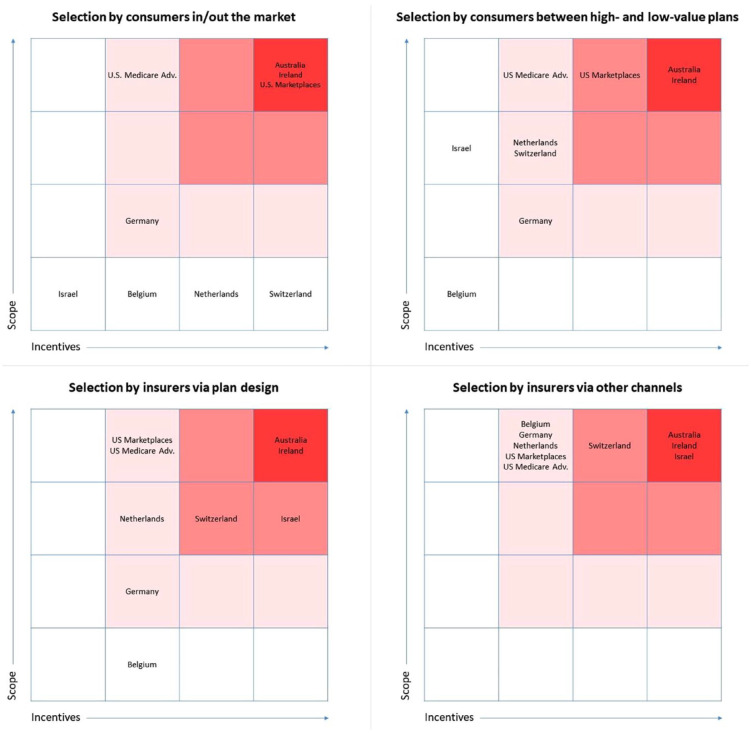
Scope and Incentives for Four Types of Risk Selection in Nine Systems.

Figure 1 contains a visual comparison of the scope and incentives for risk selection in the nine systems of interest. More specifically, each of the four diagrams classifies the nine systems along the dimensions of “scope” (vertically) and “incentives” (horizontally). In line with our conceptual framework, a distinction is made among the four types of selection, using one diagram for each.

The diagrams can be interpreted as follows. In systems with no/low scope and no/low incentives for a specific type of risk selection, the potential for that selection type to occur is very limited. The same is true for systems with either no/low scope or no/low incentives, as both the ability and the reason to select must be present to expect it to occur. In systems with both substantial scope and substantial incentives for a specific selection type, the potential for that selection type to occur is also substantial. Classifications in the diagrams composing Figure 1 do not indicate the magnitude of selection potential other than a qualitative ranking. Therefore, the relevance of our comparison is to be found in (a) the relative position of the nine systems (rather than some absolute distance between systems) and (b) the variation in patterns across diagrams (i.e., selection types). The next four subsections discuss the four diagrams in more detail.

### Selection by Consumers “In and Out of the Market”

In the four countries with a strong mandate (Belgium, the Netherlands, Israel, and Switzerland) the potential for selection by consumers in and out of the market is limited. Although the Dutch scheme—and even more so the Swiss scheme—includes “incentives” for this type of selection (since community-rated premiums exceed expected spending under the insurance contract for low risks), the mandate prevents these incentives from affecting system performance.

In U.S. Medicare, Medicare Advantage plans are more generous in dimensions of cost sharing but less generous in terms of network breadth and utilization management. Consumers can switch between Traditional Medicare and Medicare Advantage during an annual open enrollment period, leaving scope for selection in and out of Medicare Advantage. Incentives for this type of selection, however, are mitigated by the fact that premiums are heavily subsidized (which limits premium differences between Medicare Advantage and Traditional Medicare); incentives for consumers to select between the two schemes mainly result from the above-mentioned differences in coverage design. Newhouse et al. (2015) found that selection by consumers between Traditional Medicare and Medicare Advantage, net of risk adjustment, has been largely but not entirely eliminated with the current payment model.

The U.S. Marketplaces, Australia, and Ireland do not include a (strong) insurance mandate, implying substantial scope for selection in and out of the market. In the U.S. Marketplaces and Ireland, incentives for this type of selection are mitigated by risk rating according to age and consumer subsidies. In Australia, premiums are community-rated. Consequently, for young and healthy Australians, premiums might (far) exceed expected spending under the insurance. Australia fully relies on subsidies and penalties to encourage insurance uptake in this group. It is challenging to assess the net effect of premium regulation, subsidies, and tax measures in the three schemes; we therefore decided to cluster these three schemes in the same (top-right) cell.

In German sickness funds, selection in and out of the market can occur when eligible people choose PHI instead of sickness fund insurance (i.e., high-income employees, self-employed, and civil servants). Since premiums of PHI plans are risk-rated (whereas premiums of sickness fund insurance are community-rated), low-risk people in this group have some incentive to opt for PHI, while high-risk people have some incentive to opt for the sickness fund insurance.

### Selection by Consumers Between High- and Low-Value Insurance Plans

When it comes to selection by consumers between high- and low-value plans, scope depends on the extent to which plans differ (e.g., in terms of benefits covered, cost-sharing schedules, and provider network) and incentives depend on the premium structure across plan types. In Belgium plans are highly standardized, implying no/low scope; moreover, in the absence of variation in plan types, there can also be no incentives for consumers to select between plan types.

In Israel, plans differ in terms of provider network and managed care, implying scope for risk selection between high- and low-value plans. Due to the absence of premiums, however, incentives for this type of selection are limited. The absence of premiums does not necessarily mean that consumer sorting is efficient in Israel; due to selection on the side of insurers (see Sections “Selection by Insurers Via Plan Design” and “Selection by Insurers Via Other Channels”), consumers might be inefficiently distributed among plans.

In Germany, plans can differ in terms of voluntary deductibles, implying some scope for selection between high- and low-value plans. In Switzerland and the Netherlands, plans can also differ in terms of managed care, provider network, and coverage for out-of-network spending. In the U.S. Marketplaces, Medicare Advantage, and the schemes in Ireland and Australia the scope for this type of selection is even bigger since plans in these schemes also vary by cost-sharing schedules (all four) and benefits covered (Ireland and Australia). In addition to “scope,” these schemes also include “incentives” for selection between high- and low-value plans. The reason is twofold. First, premiums do not (fully) vary with expected spending. For example, the “premium rebate” for voluntary deductibles in Germany, the Netherlands, and Switzerland is community-rated per plan meaning that high-deductible plans are more attractive for low-risk people than for high-risk people. Second, and related to the first reason, the segmentation of low- and high-risk people into low- and high-value plans, respectively, exacerbates selection incentives since premium differences among plan types are magnified by variation in “health” across plan populations. For example, the premium rebates for voluntary deductibles increase as more low-risk people sort into these plans, which can exacerbate the selection between high- and low-deductible plans. In the U.S. Marketplaces, Medicare Advantage, the Netherlands, and Germany, however, this second effect is largely mitigated by sophisticated risk equalization. More specifically, risk equalization largely compensates insurers for health-related spending variation across plan populations. In Ireland and Switzerland, risk equalization is less sophisticated, but supplemented by extensive risk sharing. Australia has no risk equalization and only relies on risk-sharing arrangements.

### Selection by Insurers Via Plan Design

In all nine systems, insurers face (some) “incentives” for risk selection. The reason is that—from the perspective of insurers—revenues for insurance contracts (i.e., premiums plus risk equalization/sharing payments) are not perfectly aligned with the expected costs of these contracts. Consequently, people in good health are expected to be profitable, while those in poor health are expected to be unprofitable. In Belgium, Germany, the Netherlands, U.S. Marketplaces, and Medicare Advantage, however, these predictable profits and losses are substantially mitigated by a sophisticated risk equalization system (and in some of these systems also by risk rating according to age and region). Switzerland (with moderately sophisticated risk equalization) and Ireland (with relatively limited risk equalization) also use risk sharing. Australia relies only on (extensive, though incomplete) risk sharing, while Israel only uses limited risk equalization. Based on the qualitative description of the risk equalization and risk-sharing mechanisms in the nine countries (Table 6), we hypothesize that in relative terms the incentives for insurers to engage in selection are largest for Australia, Ireland, and Israel, followed by Switzerland. The magnitude of the differences in incentives across schemes remains an empirical question.

In terms of “scope” for selection via plan design, huge differences exist among countries. While insurers in Belgium and Germany have no or only limited flexibility regarding plan design, insurers in Australia and Ireland can differentiate their plans in many ways (e.g., in terms of benefits covered, cost-sharing schedules, provider network, and coverage for out-of-network spending).^11^ Although the U.S. Marketplaces and Medicare Advantage implemented relatively standardized tiers of benefits, insurers still have considerable flexibility with respect to cost sharing, provider network, provider fee schedules, and coverage of out-of-network spending. In Switzerland, the Netherlands, and Israel, flexibility for insurers mainly applies to provider network design and coverage of out-of-network spending. Given these differences in scope for selection via plan design, “incentives” for this type of selection are much more problematic in some countries (e.g., Australia, Ireland, and Israel) than in others (e.g., Belgium and Germany).

### Selection by Insurers Via Other Channels

As discussed in Section “Selection by Insurers Via Plan Design,” selection incentives for insurers result from mismatches between insurers’ revenues and expected costs of insurance contracts. This implies that incentives for “selection via other channels” are more or less similar to incentives for “selection via plan design.” Therefore, the classification of systems on the horizontal axis in the bottom-right diagram mimics that in the bottom-left diagram. The difference between these diagrams appears in the vertical dimension (scope): whereas the scope for “selection via plan design” is limited in some systems, the scope for “selection via other channels” is substantial in all systems. Important “other tools” for insurers include marketing and customer service, which are only moderately regulated in all nine systems. In Australia, Belgium, the Netherlands, Israel, and Switzerland, there is a strong (perceived) link between supplementary insurance and basic insurance, which provides insurers with another tool for risk selection: by making supplementary plans relatively (un)attractive to people who are predictably (un)profitable for the basic plan, insurers can engage in “cream skimming” for the basic plan. Even in systems with highly standardized basic plans, insurers have substantial flexibility when it comes to the coverage, pricing, and/or underwriting of supplementary plans. In Australia, Germany, Ireland, Israel, the Netherlands, the U.S. Marketplaces, and U.S. Medicare, there are moderate or strong links between insurers and providers of care, which enables insurers to transmit their selection incentives to providers, such as by discouraging enrollment of or service provision to unprofitable patient types. Despite differences in the “link between insurers and providers of care” and the “link between basic and supplementary plans,” the three bottom rows of Table 5 lead us to the conclusion that, overall, the scope for “selection by insurers via other channels” is substantial in all nine systems.

## Discussion

This paper has presented a conceptual framework for analyzing the “scope” and “incentives” for risk selection in health insurance markets with regulated competition. The framework distinguishes four selection types: (a) selection by consumers in and out of the market, (b) selection by consumers between high- and low-value plans, (c) selection by insurers via plan design, and (d) selection by insurers via other channels such as marketing, customer service, and design, pricing, and underwriting of supplementary insurance products. For each selection type, we have identified the health insurance system features determining scope and incentives. The second part of the paper has applied the framework to nine existing health insurance markets with regulated competition in Australia, Europe, Israel, and the United States. Our findings reveal substantial differences in scope and incentives across systems and across selection types. This section highlights our key observations and implications for health policy and research.

Our framework helps us understand why some health insurance systems suffer more from specific selection problems than others. For example, “selection by consumers in and out of the market” has been a major topic in the U.S. Marketplaces, Ireland, and Australia, but is not really an issue in systems with a strong mandate and/or low premiums due to high levels of external financing such as Belgium, Israel, Germany, the Netherlands, Switzerland, and U.S. Medicare Advantage. In the latter systems, the main concern is “risk selection by insurers.” This might also explain why European research tends to focus on risk selection (incentives) on the side of insurers (e.g., Brammli-Greenberg et al., 2018; Schmid et al., 2018; Schokkaert et al., 2018; van de Ven et al., 2013, 2007; van Kleef et al., 2018; Wasem et al., 2018), while U.S. research examines both selection by insurers (e.g., Geruso et al., 2019; Glazer & McGuire, 2000) and selection issues on the side of consumers (e.g., Geruso et al., 2023; Geruso & Layton, 2017; Newhouse, 2017; Saltzman, 2021).

Our framework makes an explicit distinction between “scope” and “incentives” for risk selection. Scope is determined by the “actions” that insurers and consumers can take, and incentives are driven by “unpriced risk heterogeneity” (a concept with different meanings on the side of insurers and the side of consumers). The relevance of this distinction is that selection “potential” requires the presence of both scope and incentives. With one of these ingredients missing, selection potential will be absent. This can be illustrated with our analysis of “selection by insurers via plan design”: although the basic health insurance schemes in Belgium and Germany include incentives for selection by insurers, the scope for selection via plan design is very limited (since plans are highly standardized). The other systems are characterized by both scope and incentives, a dangerous combination creating potential for selection via plan design. Our analysis distinguishes between two general directions for mitigating selection problems: (a) limiting selection incentives (i.e., mitigating unpriced risk heterogeneity) and (b) limiting selection scope (i.e., reducing choice options for consumers and/or tools for insurers regarding the design and marketing of plans).^12^ From an economic perspective, the first direction might be preferred over the second. The reason is that a reduction in “scope for selection” might also imply a reduction in “scope for efficiency,” given that many instruments for selection (e.g., consumer choice options and insurer tools for managed care) are also tools that can be used for enhancing efficiency. Moreover, our analysis implies that, in systems with great scope, mitigation of incentives is more important than in systems with no/low scope. For example, systems in which insurers have substantial flexibility regarding plan design (e.g., U.S. Marketplaces) might require better risk equalization models than systems with highly standardized plans (e.g., Belgium).

Our framework makes a distinction among four selection types. For two reasons, this distinction is important when analyzing selection problems in health insurance systems. First, each of these selection types comes with specific welfare effects. For example, selection by consumers can lead to underinsurance of low-risk people as well as affordability problems (particularly for low-income people) due to upward premium spirals. Selection by insurers on the other hand can lead to inefficient plan design such as suboptimal cost-sharing schedules or provider networks. For researchers analyzing selection in health insurance systems, it is crucial to be clear about the type(s) of selection focused on since different welfare effects require different welfare metrics and interpretations. Being clear about the types of selection of interest is also important for policymakers: some welfare effects might be considered more important than others, implying that some selection types might be considered more problematic than others.

The second reason for making a distinction among the four selection types is that system features (and policy instruments) affect the scope and/or incentives for these selection types in different ways. For example, an insurance mandate counteracts selection by consumers in and out of the market but might exacerbate selection by consumers between high- and low-value plans (Geruso et al., 2021). And plan payment tools such as risk equalization and risk sharing reduce selection incentives for insurers, but not necessarily for consumers (van Kleef, McGuire, et al., 2019). This implies that solving selection problems generally requires a blend of policy instruments. The framework presented in this paper might help regulators find the “right” blend for their setting of interest.

There is only one policy remedy that—in theory—mitigates all four selection problems simultaneously: risk rating of premiums to consumers. Risk rating inherently reduces unpriced risk heterogeneity (i.e., selection incentives), both on the side of insurers and that of consumers. With complete risk rating, incentives for risk selection would be absent, implying no potential for each of the four selection types, independent of scope.^13^ For reasons of fairness, however, all nine health insurance systems analyzed in this paper, rely heavily on premium regulation to limit risk rating. Without such regulation, premiums might be unaffordable for people in poor health with high expected spending. Although alternative tools are available for promoting the affordability of health insurance coverage (such as risk-adjusted vouchers or other forms of subsidies to consumers), the nine health insurance systems analyzed in this paper have not (yet) considered (refined) risk rating as a realistic policy direction. Instead, regulators of these systems have focused on other tools to solve selection problems, under the constraint of premium regulation. Apart from fairness considerations, risk rating might come with practical limitations. First, “complete” risk rating is unrealistic since consumers are likely to have more information than insurers. Second, refined risk rating might reduce the transparency of insurance plans/markets.

In the presence of premium-rate restrictions, risk equalization (possibly supplemented with risk sharing) will be necessary to mitigate selection incentives for insurers (i.e., selection types 3 and 4 in our conceptual framework) and to compensate plans for sorting of risk types into specific plans (i.e., selection type 2).^14^ Our international comparison shows substantial differences in the spectrum of risk adjusters used in the nine systems. The most sophisticated risk equalization models are found in the Netherlands, Germany, and the United States. Recent studies have shown, however, that even these sophisticated models do not completely compensate for predictable variation in health care spending (e.g., McGuire et al., 2021; van Kleef, Eijkenaar, et al., 2019; Zink & Rose, 2021). More specifically, these studies show that—*net of* risk equalization—some risk types are predictably profitable for insurers, while others are predictably unprofitable. Improvements in risk equalization systems are required to further reduce selection incentives for insurers. However, this is easier said than done since the development and implementation of additional risk adjusters come with complex tradeoffs. For example, adding diagnoses-based risk adjusters may not only reduce selection incentives but can also increase incentives for gaming and reduce incentives for cost control. To help policymakers make these tradeoffs, further research is needed to quantify the incentive effects of payment systems. While for some systems, such as the Netherlands, Switzerland, and the United States, there is a growing empirical literature on the performance of risk equalization models (e.g., Kauer et al., 2020; McGuire et al., 2021; van Kleef, Eijkenaar, et al., 2019; Zink & Rose, 2021), for other systems, such research is still limited.

As was already mentioned above, different selection types might require different metrics for quantifying the incentives and/or welfare effects of risk selection. In addition, we believe that it is important to align the choice of metric(s) for a specific selection type with the “scope” for that selection type. In other words: the choice of metrics should follow from the “actions” consumers and/or insurers can take in the health insurance system of interest. This has the important implication that, in terms of selection metrics, there is no one-size-fits-all metric. Specific settings will require specific measures dependent on the scope for risk selection. In Belgium, for instance, where plans are highly standardized, it makes little sense to evaluate selection incentives with metrics for service-level selection. Instead, it is more relevant to apply metrics related to marketing actions, for example, predictable profits/losses for groups that are potential targets of such actions. In systems with scope for selection via both design and marketing of insurance plans, both types of metrics are relevant. Moreover, the operationalization of metrics for service-level selection and selection via marketing should be in line with the spectrum of possible insurer actions. In general, more flexibility for insurers will imply more sophisticated metrics. For example, the operationalization of selection metrics will be different in systems where insurers can target 10 specific groups and differentiate among 10 specific services than in a system where insurers can target 100 specific groups and differentiate among 100 specific services. For further discussion of actions and metrics, see Layton et al. (2017).

In this paper, we have analyzed the scope and incentives for risk selection by looking at specific system *features*. We realize that there might be other features that explain why specific types of risk selection are more problematic in some systems than in others. One potentially important factor is consumer behavior. Consumers do not always choose the insurance plan that provides them with the highest value over costs. Reasons for suboptimal choices can be unavailability of (comprehensible) information about the price and quality of insurance plans, hassle and search costs, and behavioral factors related to inertia (Handel & Kolstad, 2015). Selection by consumers is expected to be greater in systems where consumer response to differences in insurance plans is stronger (Handel et al., 2019). Moreover, stronger consumer response comes with larger expected benefits from selection actions by insurers and thus increases incentives for insurers to engage in these actions. Although consumer behavior and the availability of information are likely to vary across systems, these dimensions are hard to grasp. The same is true for other potentially important factors such as the strength of reputation effects, the strength of competition, and the attitudes and beliefs toward equity. The latter might affect both what regulations are feasible and how strongly individuals and insurers strive to take advantage of insurance programs for personal gain. Expanding our framework with these and other relevant determinants of scope and incentives for selection is an important direction for future research.

## Notes

1. Risk selection can include actions that intend to exploit unpriced risk heterogeneity, but eventually do not. For example, when all insurers in a mandatory health insurance scheme engage in actions to avoid sick people (e.g., by not contracting the best doctors and/or providing poor customer service), they might be equally successful with these actions. Consequently, no unpriced risk heterogeneity is exploited, and pooling arrangements remain intact. Nevertheless, these actions can have important negative effects such as the non-availability of high-quality health insurance plans and reduced access to high-quality care (van de Ven et al., 2017).

2. Risk selection can also include actions that do not explicitly *intend* to exploit unpriced risk heterogeneity, but eventually do. In general, any correlation between unpriced risk heterogeneity and consumer preferences regarding plan characteristics (e.g., benefits covered, level of cost sharing, and provider network) can lead high- and low-risk consumers to sort into different plan types, irrespective of the underlying intentions.

3. Note that a fifth type of selection, i.e., selective underwriting by insurers, is not considered here. “Open enrollment” provisions (i.e., the requirement for insurers to accept any applicant irrespective of her characteristics) prohibit selective underwriting in all nine systems included in our comparison.

4. In this paper, both “efficiency” and “fairness” are used as multidimensional concepts. For example, efficiency can relate to “allocative efficiency” (e.g., consumers select the insurance plan that provides them with the highest value over money) and “efficiency in production” (e.g., insurers maximize value for money). Fairness can relate to ideas about “equity in financing” (e.g., richer contribute more than the poor, and the sick pay no more than the healthy, principles that Europeans refer to as “solidarity”) and ideas about “equity in access” (e.g., access according to medical need, not ability to pay). In our considerations below, we will describe the relevant efficiency and fairness effects as specifically as possible.

5. Notably, there is no single premium difference between the more and less generous plan that leads to the efficient sorting of consumers between plans (Glazer & McGuire, 2011). Efficient sorting takes place when each consumer faces her incremental marginal cost at the more generous plan. One incremental price may convey the right incentives to the average enrollee but is incapable of pricing fully efficiently due to heterogeneity in health risk.

6. The difference with type-3 selection actions is that type-3 actions are about explicit insurance plan features (e.g., in the form of barriers to care), while “instructing/incentivizing health care providers to encourage expensive patients to switch to another insurer or plan type” is about persuasion.

7. The German system finds itself in between these extremes, as will be explained in our analysis later on.

8. In case of a fine, an insurance mandate can also be seen as a system characteristic affecting the *incentives* (rather than *scope*) for risk selection. For example, a higher fine increases the cost of being uninsured which reduces the incentives for selection out of the market. Since penalties for violating an insurance mandate are not necessarily defined in terms of a monetary fine, however, we classified this system characteristic under *scope*.

9. No firm with financial responsibility, for profit or not for profit, can withstand repeated losses, except when insurers know that the government or other parties will step in to help cover losses. Also, firm (and manager) success is tied to the surplus of revenue over cost. Positive net revenue can fund various insurer initiatives and may be positively related to managerial compensation. These observations justify our assumption that in any competitive health insurance market, insurers with financial responsibility will to some extent be sensitive to selection incentives. Nevertheless, we acknowledge that the organizational form of insurance companies might impact the strength of insurers’ response to selection incentives.

10. Recently (in 2022), it has been decided to give up the whole system of individual financial responsibility and to move toward a situation of “collective” responsibility of all the sickness funds. While the specific modalities of this new system have not yet been determined, the financial risks for the individual sickness funds will certainly become much smaller—or even nil. Our analyses for this paper are based on the situation in Belgium until 2022 (i.e., do not consider the proposed reform toward collective responsibility).

11. In Ireland, product proliferation has become increasingly significant with currently >300 products.

12. An interesting recent example of how scope for selection can be reduced comes from Switzerland. Until recently, Swiss insurers used extraordinarily high and differentiated commissions for intermediaries to let these intermediaries attract good risks for their insurance products. This resulted in an inefficient Nash-equilibrium with too large commissions because each insurer who reduced its commission would risk the good risks to leave to competitors who pay higher commissions. Recently, the insurer associations signed a treaty that banned high commissions, which came into effect on January 1, 2021. The insurers established a commission to monitor the compliance with this treaty. This substantially reduced the scope for selection by insurers via this particular channel (intermediaries).

13. Another advantage of allowing some degree of risk rating is that consumers will be incentivized to avoid health problems and maintain spending in the event of a health problem (to avoid reclassification to a higher premium category). This will only be welfare improving, however, if consumers selectively avoid *unnecessary* health care. As soon as consumers start avoiding necessary care (which is also referred to as underconsumption) the net welfare effects of risk rating are unclear. Moreover, when underutilization is already present in the system (even without risk rating), prior utilization might be an imperfect indicator of health. For example, when a consumer has no prior spending, is that because the consumer is in good health or because the consumer cannot afford to go to the doctor? Similar problems arise when using utilization-based health indicators as risk adjusters in risk equalization models.

14. As indicated in Table 4, risk equalization does not reduce incentives for selection by consumers in and out of the market (i.e., selection type 1 in our conceptual framework). When risk equalization payments are (partly) financed internally—meaning that the risk equalization fund is financed with premium revenues from insurers—risk equalization can even exacerbate incentives for low-risk consumers to disenroll (or not enroll). The reason is as follows: with internal risk equalization, insurers pay a contribution for low-risk people to the risk equalization fund. (The regulator uses these contributions to finance payments *to* insurers for high-risk people.) From the insurers’ perspective, this increases the cost for low-risk people (i.e., claims cost + risk equalization contribution) and most likely also the premium for plans with a disproportionate share of low-risk people (e.g., high-deductible plans and narrow-network plans). Consequently, some of the low-risk people in these relatively cheap plans might not be willing to pay the higher premium and—in the absence of a strong mandate—decide to disenroll.
